# Medical Research; American Red Cross Commission's Report

**Published:** 1919-01-25

**Authors:** 


### TRENCH FEVER.

### Medical Research; American Red Cross Commission's Report.

(PUBLISHED AT THE OXFORD UNIVERSITY PRESS, 1918.)

OF the many publications now appearing and bringing before the medical world the intensely interesting details of the vagaries and novelty of disease in war none is more admirable and deserving of widespread appreciation than the Report on Trench Fever by the American Commission. This volume is issued in beautiful style by the Clarendon Press, with excellent, clearly described diagrams, charts, and photographic illustrations, while the wealth of detail is inserted in such a precise and readable manner that the least specialised in epidemic disease may enjoy it to the utmost. Its scientific value is at once apparent, and, as this report may well become a classic on the subject, it is hoped that it may claim the attention, not of medical men only but of many members of the general public.

In the early stages of the war, and likely now at its conclusion momentarily to revive, came a marked tendency to introduce at home a new set of vague but accepted diagnoses and a whole train of "fashionable" diseases. At one time trench fever stood in great danger of being adopted as one of these, and the reading of at least the major conclusions of the Commission should do much to counteract this failing. The often ludicrous ideas, such as find their way into the daily press, of its possible relationship to rheumatism, gout, or the modern "influenza" are at once dispelled, and the mode of spread—entirely through the activities of body lice—makes it more than distasteful as a ready-to-hand diagnosis.

In the first place, the specific nature of the fever is established. One great problem remained. Was trench fever a modified typhoid or paratyphoid? Here the Commission began its work, and soon established three main points—namely, (1) that no abnormal bacterial growth was obtained from the blood, stools, or urine; (2) that no bacterial substances affecting the growth of the typhoid group were ever obtained from trench fever blood; and (3) that Dreyer's agglutination method showed no indication of the typhoid organisms in or upon the patients. The conclusion is therefore drawn that relationship to the enteric disease is excluded, and that trench fever is due to a specific virus.

The general symptomatology is well known, but well worth reading as set forth in the report. The various types are here excellently classified, and that all varieties are but different manifestations of the same disease was shown in their experimental transmission. The type appearing in the inoculated case did not always conform to that of the original patient.

The next step gave the character of the virus itself. In summary, this substance resists heat considerably, but is destroyed in thirty minutes by a moist temperature of 70° C.; it is filterable, and is found in the blood plasma, the urine, and often in the saliva of infected subjects. Careful and elaborate inoculation experiments provided this link in the chain. Then, finally, in all ways making most fascinating reading, comes the determination of the mode of spread. Acting upon the idea that one of the many varieties of insects infesting the troops was the intermediate host of the virus, experiments were made with the common louse. The disease was transmitted by means of a fresh, experimentally infected strain of parasite, and also by those caught on the bodies of infected men in the trenches.

Throughout the Commission worked as a team, and a strong one. Temporary accommodation at a relatively short distance behind the firing-line—so short, in fact, that the German advance of March last caused a hurried evacuation and removal to Neuilly—did not add to the ease and convenience amongst which they completed the work, and the picture of the eighty-two volunteers from the American Army who submitted to inoculation, well knowing the risk they ran, and who fed lice in specially constructed cells upon their arms for many consecutive days, is characteristic of the spirit of enterprise and self-sacrifice with which the whole labour was carried out. The resulting complete co-ordination of results is more than deserved.

Turning, in conclusion, to the methods of limiting and eradicating disease, the Commission give one last convincing proof of the value of their work. The insect was found to convey the disease not by its bite alone (the usual method), but also by contact of its infected excrement with any abraded surface on the human body. Also a man may be entirely free from lice at the time he typically develops trench fever, since the parasite need only remain upon the individual for a short time to produce infection. Of the patient's urine and sputum thorough sterilisation is advised, as both may be infected. In the last paragraph of their concluding summary so concise an indication of methods of limitation is given that the words of the Commission may be quoted in full:—

> On account of the great importance of the matter the following sanitary regulations are advised: Exceedingly great care should be taken to completely disinfect all patients as soon as practicable, and particularly upon their entering the hospital. Patients on entrance should be carefully bathed, and subsequently sponged with alcohol. Their clothing and blankets should be removed, and, whether or not lice or ova found upon them, should be carefully sterilised by moist heat at a temperature not below 70° Centigrade for half an hour, since it is possible for the virus to be still present on the clothing. It should be borne in mind that a man with trench fever may be entirely free from lice at the time that he develops symptoms of the disease. Trench fever patients should at all times be carefully protected from louse infestation, and inspection of them for lice should be made daily. They should be treated in separate wards. As the urine contains the virus and is infective it should be sterilised during the active stages of the disease. Sputum cups should be provided for patients, and any expectorated sputum and saliva from them sterilised. Officers should regard the systematic destruction of lice as one of the most urgent of their duties, and should exercise every effort to prevent louse infestation among soldiers and to see that

**Trench Fever**—(*continued*).

any of them infested with lice are promptly disinfected and their clothing sterilised.

The range and prevalence of lousiness and itch have been one of the surprises of the Great War, and, although we at home had come to think this country tolerably free, this report comes as a forcible reminder of the potential capabilities of the louse. Fortunately, it also suggests proved counter-measures. In this direction, from early times, the Allied authorities had done much to control one of the soldiers' greatest trials, and the American Red Cross have ably and notably supplemented their efforts.

To Major Richard P. Strong, Med. Res. Corps, A.E.F., the Editor, and his excellent colleagues the warmest congratulations must be offered.

